# Pharmacokinetics of Siddha Antidiabetic Polyherbal Formulation Madhumega Chooranam in Healthy Volunteers

**DOI:** 10.7759/cureus.72348

**Published:** 2024-10-25

**Authors:** Chandrasekaran Anbarasi, Sadagopan Thanikachalam

**Affiliations:** 1 Siddha Central Research Institute, Central Council for Research in Siddha, Chennai, IND; 2 Cardiology, Sri Ramachandra Institute of Higher Education and Research, Chennai, IND

**Keywords:** diabetes, gallic acid, pharmacokinetics, polyherbal formulations, siddha

## Abstract

Background

Madhumega Chooranam (MMC), a traditional Siddha polyherbal formulation, is used for diabetes management. Understanding its pharmacokinetics is crucial for evaluating its efficacy and safety in clinical practice. This study aimed to assess the pharmacokinetics of gallic acid, a key component of MMC.

Methodology

Ten healthy volunteers were selected based on their willingness to participate and the absence of significant clinical conditions, including liver, kidney, heart, peripheral nerve disorders, or allergies. During the screening phase, participants with abnormal laboratory results in blood chemistry, hematology, or urine analysis were excluded. All participants provided written informed consent before the initiation of the study. Participants were administered a single oral dose of 6 g of MMC. Blood samples were collected at predetermined intervals over 24 hours to estimate gallic acid content used as a pharmacokinetic marker.

Results

Gallic acid concentration peaked in some participants’ plasma samples at varying intervals post-administration, indicating individual differences in absorption and metabolism. In contrast, no gallic acid peaks were detected in the plasma samples of three participants, suggesting potential variability in metabolic response or rapid clearance.

Conclusions

The study’s findings underscore the complexity of MMC’s pharmacokinetics, influenced by individual metabolic and genetic factors. Additionally, this research highlights the need for an interdisciplinary approach, integrating traditional medicine with modern pharmacogenomics and gut microbiota studies, to fully understand Siddha formulations’ pharmacokinetics and therapeutic potential. Future studies should focus on larger sample sizes and consider genetic and dietary factors to enhance the understanding of MMC’s efficacy and safety in diverse populations.

## Introduction

The Siddha system of medicine is an ancient traditional medicine practiced primarily in Tamil-speaking regions and countries. Madhumega Chooranam (MMC), a polyherbal formulation consisting of seven herbs, namely,* Terminalia chebula*, *Phyllanthus emblica*, *Murraya koenigii*, *Tinospora cordifolia*, *Cyperus rotundus*, *Syzigium cuminii*, and *Phyllanthus amarus*, is routinely prescribed in the Government of Tamil Nadu hospitals for several decades to control type 2 diabetes. A population study of urban, rural, and semi-urban regions for the detection of endovascular diseases and prevalence of risk factors and holistic intervention study (PURSE HIS) was conducted in 2008-2018 at Sri Ramachandra University, Chennai, funded by the Department of Science and Technology, Government of India. This study was conducted to understand the prevalence and progression of subclinical and overt endovascular disease (EVD) and its risk factors in urban, semi-urban, and rural communities in South India. It was designed to generate clinical evidence for effective, affordable, and sustainable community-specific intervention strategies to control risk factors for EVD. This study included MMC as a holistic intervention for controlling type 2 diabetes [1]. PURSE HIS conducted preclinical safety studies, including acute, 28-day repeated oral toxicity, 90-day chronic toxicity evaluations, and efficacy studies on MMC. It also conducted Geno geno-toxicological tests, such as the in-vitro chromosomal aberration test in cultured human lymphocytes and the in-vivo micronucleus test in Balb/c mice. It ensured the safety of the MMC. Additionally, a clinical study demonstrated that MMC intervention reduced blood sugar levels [2]. The increasing use of herbal medicines and traditional herbal products emphasizes the need to evaluate quality, efficacy, and safety in compliance with good manufacturing and agricultural practices [3]. Standardizing traditional herbal products, proving their content and efficacy, determining their pharmacokinetic properties and bioavailability, establishing dose-response relationships, and identifying interactions with conventional drugs are highly warranted. Similar to all traditional medicines, Siddha formulations face constant challenges, such as standardization and pharmacokinetic profiling (absorption, distribution, metabolism, and excretion) of biomarkers. It has been subjected to systematic preclinical, clinical safety, and efficacy studies [2,4]. However, until now, no pharmacokinetic profiling or bioavailability assessment of MMC has been performed.

Safe and effective drug treatment is not only a function of the physical and chemical properties of drugs but also a function of how the human body responds to the administration of medication. The study of the bodily processes that affect the movement of a drug in the body is referred to as pharmacokinetics. Therefore, the course of drug action is directly correlated with the concentration of the drug in the bloodstream and depends upon the absorption, distribution, metabolism, and excretion processes. Assessing the bioavailability of traditional medicines is crucial to understanding their effectiveness and potential therapeutic benefits. In addition, pharmacokinetic assessment helps to identify essential compounds and develop innovative formulation approaches to improve their bioavailability. Pharmacokinetic profiling of polyherbal drugs presents several challenges owing to the complexity of the components in the formulations and their active principles. Factors such as solubility, permeability, and metabolism can influence the variability in the pharmacokinetics of individual herbs within a polyherbal formulation. Hence, a bioactive-guided pharmacokinetic approach is required to determine the pharmacokinetics of relevant markers in formulations with many markers [5].

Free radical production is a continuous and inevitable process of normal metabolism. Due to internal or external factors, the overproduction and accumulation of reactive oxygen species create an imbalance leading to oxidative stress, which increases the production of superoxide radicals and hydrogen peroxide. In the presence of suitable transition metal catalysts, the reactive species, the free radicals, further interact to form highly toxic hydroxyl radicals. Naturally occurring antioxidants can protect cells from oxidative stress via many pathways. Polyphenols form an important class of naturally occurring antioxidants that are vital in decreasing the incidence of atherosclerosis, diabetes, and neurodegenerative diseases [6]. Among various polyphenols, gallic acid is a naturally occurring low-molecular-weight triphenolic compound revealed as a strong antioxidant in many studies. A preclinical study in diabetic rats showed that the phenolic fraction of MMC improved carbohydrate metabolism and attenuated oxidative stress in rats with type II diabetes. Thus, we selected gallic acid as one of the key marker compounds for understanding the pharmacokinetics of MMC. The primary objective of this study was to evaluate the pharmacokinetics of gallic acid in human plasma after oral administration of the Siddha polyherbal formulation MMC. Additionally, the study aimed to explore the influence of factors such as pharmacogenomics, gut microbiota, and dietary interactions on these pharmacokinetic patterns.

## Materials and methods

Study participants

The study was conducted at the Siddha Central Research Institute (SCRI) inpatient facility under the Central Council for Research in Siddha, Ministry of Ayush, Government of India, located in Chennai, Tamil Nadu, India. It included 10 healthy volunteers aged 20-40 years. Participants were selected based on their willingness to participate and the absence of significant clinical conditions, including liver, kidney, heart, or peripheral nerve disorders or allergies. One week before the start of the study, the volunteers were screened using modern and Siddha clinical examinations, vital parameter monitoring, medical history, and demographic information. Siddha Eight-Fold Examinations include *Naa* (tongue examination), *Niram* (color examination), *Mozhi* (speech examination), *Vizhi* (eye examination), *Naadi* (pulse examination), *Sparisam* (touch examination), *Malam* (stool examination), and *Moothiram* (urine examination). This evaluation helps diagnose the balance of *Vali*, *Azhal*, and *Iyam*, which governs physical and mental well-being. Laboratory tests, including fasting blood sugar, two-hour postprandial blood sugar, HbA1c, lipid profile, liver function test, renal function test, thyroid profile test, routine urine test, HIV test, Chest X-ray posteroanterior view, and ECG in 12 leads, were performed. Only non-smokers, non-alcoholics, and those not taking herbal medicines were enrolled. Participants with abnormal laboratory results in blood chemistry, hematology, or urine analysis were excluded during the screening phase. All participants provided written informed consent before the initiation of the study.

Drug administration

Following a 10-hour overnight fast, a single oral dose of 6 g of MMC (a polyherbal powder), which consists of seven herbs (T*erminalia chebula*, *Phyllanthus emblica*, *Murraya koenigii*, *Tinospora cordifolia*, *Cyperus rotundus*, *Syzigium cuminii*, and *Phyllanthus amarus*), was given. The dietary restriction was designed to minimize external phenolic intake and control phenolic exposure. Breakfast was not provided four hours after drug administration. Instead, chapatti, bread, milk, cheese, curd, and eggs were served for lunch and dinner. Two days before the investigation, the participants were given a low-phenolic diet that excluded curry leaves, pepper, turmeric, ginger, garlic, tea, fruit, and fruit juices.

Collection of blood samples

Blood samples (2 mL) were collected in heparinized tubes at predetermined intervals (0, 0.5, 1, 2, 4, 6, 9, 12, and 24 hours). Following a 10-minute centrifugation at 3,500 rpm, the plasma was cautiously moved into airtight containers and preserved at -80°C.

Determination of gallic acid in plasma samples and pharmacokinetic analysis

Gallic acid concentrations in plasma samples were analyzed using ultra-high-performance liquid chromatography (Thermo Scientific, USA), specifically the Rapid Resolution Liquid Chromatographic System 3000 series (RRLC-3000) equipped with a 135 autosampler in the Animal and Mineral Drug Research Lab, Siddha Central Research Institute, Chennai. The analysis used a binary isocratic method with a diode array detector, and instrumental control was managed using the Chromeleon software. Gallic acid plasma concentration-time curves were generated for each participant, and pharmacokinetic variables were evaluated using the non-compartmental method. The maximum concentration (Cmax) and the time to reach maximum concentration (Tmax) were extracted directly from individual time-concentration profiles. The area under the time-concentration curve from zero to 24 hours (AUC0-24 hours) and the total area under the curve (AUC0-∞) were determined using linear and log-linear trapezoidal techniques.

The study was conducted for three days in March 2018. The Institutional Human Ethics Committee (approval number: IHEC/SCRI[CCRS]-1/2015-16/02) approved the trial. Subsequently, it was prospectively registered in the Clinical Trial Registry of India (CTRI/2017/12/010977).

## Results

The study was conducted on 10 healthy male volunteers who voluntarily consented to participate, with a mean age of 26 years. The screening parameter values of the participants were within the normal limit (Table 1). The 12-lead ECG obtained for all 10 volunteers was within normal limits. Screening for HIV showed negative results for all study participants. The study participants’ body constitution (*Udal Iyal*) was assessed based on the Siddha system of medicine. Among the 10 study participants, five (PK03, PK05, PK07, PK08, PK09) were *Azhal Iyam* type, three (PK04, PK06, PK10) were *Azhal Vali *type and two (PK01, PK02) were *Vali Azhal* type. The results showed that the study participants were not uniformly distributed among the *Udal Iyal* (body constitution/Prakriti type) (Table 2).

**Table 1:** Screening parameters of the study participants (N = 10). BMI: body mass index; BP: blood pressure; HbA1c: glycated hemoglobin; HDL: high-density lipoprotein; LDL: low-density lipoprotein; SGOT: serum glutamic-oxaloacetic transaminase; SGPT: serum glutamic-pyruvic transaminase; ESR: erythrocyte sedimentation rate; RBC: red blood cells; T3: triiodothyronine; T4: thyroxine; TSH: thyroid-stimulating hormone

Parameters	Mean	SD
Age (years)	26.10	5.47
Height (cm)	167.20	6.92
Weight (kg)	68.10	9.64
BMI (kg/m^2^)	24.25	2.06
Systolic BP (mmHg)	113.00	11.06
Diastolic BP (mmHg)	73.40	5.97
Fasting blood sugar (mg/dL)	75.40	6.17
Two-hour postprandial (mg/dL)	90.70	16.37
HbA1c (%)	5.87	0.30
Total cholesterol (mg/dL)	178.80	25.59
Triglyceride (mg/dL)	84.40	43.80
HDL-cholesterol (mg/dL)	40.70	3.50
LDL-cholesterol (mg/dL)	125.00	22.25
Blood urea (mg/dL)	19.5	0.71
Serum creatinine (mg/dL)	0.9	0.14
SGOT (IU/L)	29.0	7.07
SGPT (IU/L)	51.5	16.26
Alkaline phosphatase (IU/L)	103.0	28.28
Bilirubin (mg/dL)	0.85	0.35
Total protein (g/dL)	7.05	0.07
Albumin (g/dL)	4.5	0.28
Globulin (g/dL)	2.55	0.21
Hemoglobin (g/dL)	14.84	1.09
Total count (cells/mm^3^)	7,180	1,259.45
Polymorphs (%)	53.20	9.73
Lymphocytes (%)	43.80	17.25
Eosinophils (%)	6.95	6.89
ESR (mm/hour)	8.8	6.12
Platelets (lakhs/mm^3^)	2.58	0.47
RBC (millions/mm^3^)	5.11	0.34
Packed cell volume (%)	44.90	2.47
T3 (ng/mL)	1.36	0.22
T4 (μg/dL)	7.23	1.36
TSH (mIU/L)	1.72	1.35

**Table 2:** Gallic acid concentrations of the study participants along with the body type at various time intervals.

Study participant ID	*Udal Iyal* (body type)	Time (hours)	Area (μau)	Gallic acid concentration (μg/mL)
PK01	Vali Azhal	9	38,161	8.046
PK02	Vali Azhal	9	90,888	19.163
PK03	Azhal Iyam	1	29,861	6.296
PK04	Azhal Vali	-	-	0
PK05	Azhal Iyam	6	33,142	6.988
PK06	Azhal Vali	-	-	0
PK07	Azhal Iyam	6	42,304	8.920
PK08	Azhal Iyam	1	164,285	34.639
		6	40,410	8.520
PK09	Azhal Iyam	1	19,968	4.210
PK10	Azhal Vali	-	-	0

The retention time for the gallic acid standard in human plasma was 7.01 minutes from the method developed using high-performance liquid chromatography (HPLC). Hence, this method was used to estimate the gallic acid content in human plasma samples collected from the study participants at various intervals.

**Figure 1:**
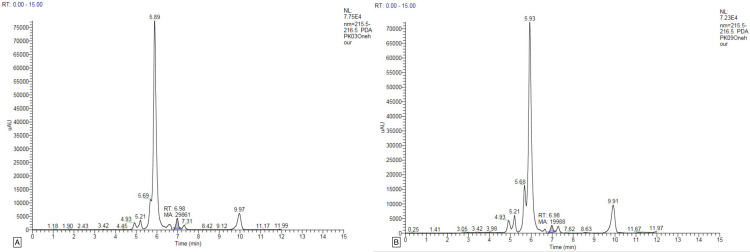
HPLC graph of gallic acid concentration after oral administration of MMC in human plasma at the first hour. A: HPLC graph of gallic acid concentration after oral administration of MMC in human plasma of PK03 at the first hour. B: HPLC graph of gallic acid concentration after oral administration of MMC in human plasma of PK09 at the first hour. HPLC: high-performance liquid chromatography; MMC: Madhumega Chooranam

**Figure 2:**
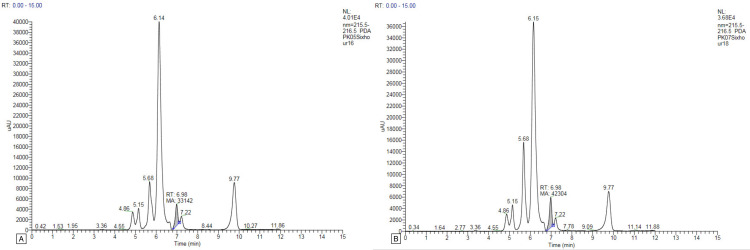
HPLC graph of gallic acid concentration after oral administration of MMC in human plasma at the sixth hour. A: HPLC graph of gallic acid concentration after oral administration of MMC in human plasma of PK05 at the sixth hour. B: HPLC graph of gallic acid concentration after oral administration of MMC in human plasma of PK07 at the sixth hour. HPLC: high-performance liquid chromatography; MMC: Madhumega Chooranam

**Figure 3:**
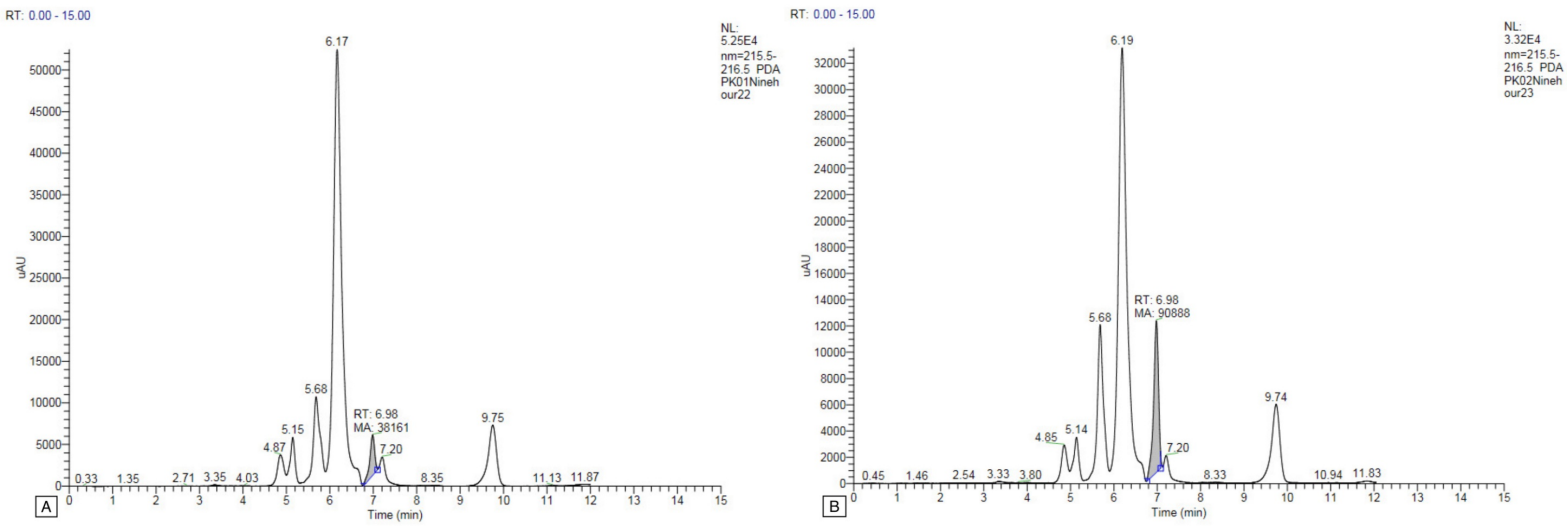
HPLC graph of gallic acid concentration after oral administration of MMC in human plasma at the ninth hour. A: HPLC graph of gallic acid concentration after oral administration of MMC in human plasma of PK01 at the ninth hour. B: HPLC graph of gallic acid concentration after oral administration of MMC in human plasma of PK02 at the ninth hour. HPLC: high-performance liquid chromatography; MMC: Madhumega Chooranam

**Figure 4:**
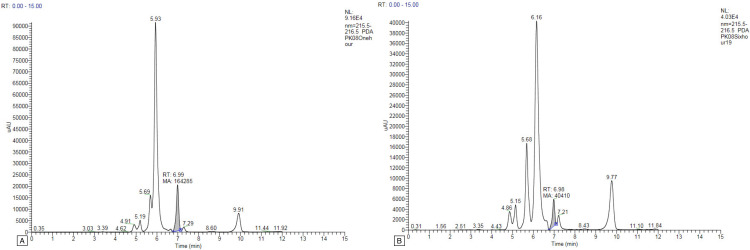
HPLC graph of gallic acid concentration after oral administration of MMC in human plasma of PK08 at the first and sixth hours. A: HPLC graph of gallic acid concentration after oral administration of MMC in human plasma of PK08 at the first hour. B: HPLC graph of gallic acid concentration after oral administration of MMC in human plasma of PK08 at the sixth hour. HPLC: high-performance liquid chromatography; MMC: Madhumega Chooranam

Only one or two peaks were found in the plasma samples of all study participants, and no peak was found at the other time intervals. The gallic acid concentration peaked in the first-hour plasma sample for two study participants (PK03 and PK09), with concentrations of 6.296 and 4.210 μg/mL, respectively (Figure 1). For two study participants (PK05 and PK07), the gallic acid peak appeared in the plasma at the sixth hour of oral administration, with 6.988 and 8.920 μg/mL concentrations, respectively (Figure 2). For two study participants (PK01 and PK02), the gallic acid peak appeared in the ninth-hour plasma sample with concentrations of 8.046 and 19.163 μg/mL, respectively (Figure 3). For the study participant PK08, the gallic acid peak appeared at two time points, in the first and sixth hour, at 34.639 and 8.520 μg/mL concentrations, respectively (Figure 4). For the remaining three study participants (PK04, PK06, and PK10), there were no gallic acid concentration peaks in the plasma samples at any time intervals collected 24 hours after oral administration of the study drug.

## Discussion

This novel attempt evaluates the oral pharmacokinetic pattern with gallic acid content as a marker in the Siddha polyherbal formulation MMC. The study showed that the appearance of gallic acid and its peak level in human plasma after MMC administration followed a different pattern than that of the standard gallic acid.

Gallic acid is a prominent dietary polyphenol constituent due to its exceptional absorbability and potent metabolic derivatives. An earlier pharmacokinetic study conducted on a single herb, *Emblica officinalis*, estimated gallic acid from its extract using HPLC to be 8.21%, and gallic acid was used as a marker [7]. It showed the maximum serum concentration (Cmax) was 4.59 ± 0.95 μg/mL in the extract form, and t1/2 was 6.0 ± 0.33 hours when compared to the standard (pure form GA) Cmax of 6.38 ± 1.08 μg/mL and t1/2 4.92 ± 0.36 hours. Another pharmacokinetic study conducted in Assam tea brew also used gallic acid as a marker, wherein after administration of 0.3 mmol GA in 200 mL tea brew showed that gallic acid was rapidly absorbed with the time of maximum serum concentration (Tmax) of 1.39 ± 0.21 hours and the highest gallium acid concentration observed in plasma (Cmax) of 2.09 ± 0.22 μmol/L. At the same time, for the standard acidum gallicum tablet, the Tmax was 1.27 ± 0.20 hours and Cmax was 1.8309 ± 0.16 μmol/L [8]. Hence, gallic acid is a valuable marker for understanding the pharmacokinetics of MMC. HPLC estimated the quantification of gallic acid present in MMC to be 6.008%. The gallic acid peak (i.e., retention time) from the plasma sample was standardized analytically using HPLC for human plasma and was calculated as 7.01 minutes.

In particular, no gallic acid peak was noted in any fasting plasma samples from the 10 human participants, confirming that gallic acid was not present in the plasma before drug administration. In addition, the individual variation of gallic acid concentration peaks in plasma at different time points (1, 6, and 9 hours) for seven study participants, while no peaks were detected in three study participants (PK04, PK06, and PK10). It is necessary to have a minimum of three values to calculate the pharmacokinetic parameters such as Cmax, Tmax, the volume of distribution (Vd), elimination half-life (t1/2), and total plasma clearance (CL). However, in practice, the study behaved differently; only one or two peak values were noted in the HPLC graph for each study participant. Hence, these parameters could not be calculated using available data. Pharmacogenomics, the role of gut microbiota in polyphenol conversion, non-polyphenolic dietary component interactions, and interactions with proteins might have interfered with the regular kinetics of the gallic acid content of the herbs in MMC.

A consistent pharmacokinetic pattern was observed for a single herbal extract of gallic acid as a biomarker from which the pharmacokinetic parameters for this study were derived [7,8]. Interestingly, the phenolic-rich fraction of MMC mediates its effects by inhibiting gluconeogenesis and activating the glycolytic pathway, improving carbohydrate metabolism, and reducing oxidative stress in type II diabetic rats [4]. Additionally, a clinical study demonstrated that MMC intervention reduced blood sugar levels [2]. Therefore, it is possible that phytochemicals, particularly other phenolic fractions found in each herb of MMC, may have affected the regular pharmacokinetics of gallic acid in this study.

Pharmacogenomics investigates how genetic variations among individuals influence their drug responses by integrating genomics, proteomics, transcriptomics, and metabolomics [9]. Genetic differences in drug-metabolizing enzymes, such as cytochrome P-450 and N-acetyltransferase, affect drug efficacy. Over 14 million single nucleotide polymorphisms scattered throughout the human genome highlight this complexity [10]. Approximately 40% of CYP-dependent drug metabolism is mediated by polymorphic enzymes, which exhibit significant interindividual activity variations primarily due to genetic factors [11,12]. External factors such as age, sex, environment, diet, and drug interactions also impact drug metabolism. Traditional medical systems, including Ayurveda, Traditional Chinese Medicine, Korean Sasang constitutional medicine, and Japanese Kampo, classify individuals based on genetic and physiological traits, emphasizing personalized medicine [13-20]. In this study, participants were categorized into three Siddha body constitution types, namely, *Vali Azhal*, *Azhal Vali*, and *Azhal Iyam*, and varying pharmacokinetic profiles for MMC were observed. For the three participants, PK04, PK06, and PK10, who were classified as *Azhal Vali*, there was no gallic acid peak in any of the plasma samples collected at various time points. Siddha literature describes that *Azhal* aids in digestion and absorption. *Vali* nourishes *Azhal* in the above digestive process and helps distribute the nutrients throughout the body. The predominance of the *Azhal* character, when combined with the *Vali* character, might have enhanced rapid metabolism (rapid metabolizer), which might be the possible reason for the non-appearance of the gallic acid peak. For the two participants, PK01 and PK02, who were classified as *Vali Azhal*, the gallic acid peak appeared in the ninth hour of the plasma sample. Siddha describes that despite a high food intake, *Vali* gets less nourishment. Considering this quality, the predominance of *Vali* character in *Vali Azhal*’s body constitution might have delayed the present study’s absorption (slow metabolizer). For the five participants, PK03, PK05, PK07, PK08, and PK09, who were classified as *Azhal* *Iyam*, the gallic acid peak appeared either in the first hour or the sixth hour or in both the first and sixth hour. Siddha literature describes the quality of *Iyam* as sluggish or slow in action. Hence, when the *Azhal* character (more active) combined with the *Iyam* character (sluggish), a gallic acid peak appeared at least at one point. These findings highlight pharmacogenomic variability and suggest that integrating Siddha body constitution knowledge with modern pharmacokinetics can enhance personalized medicine.

Herbal products undergo significant modifications due to gut microbiome enzymes, affecting their effectiveness and availability [21]. Polyphenols, often with low bioavailability, interact with the gut microbiome in the colon, where they are converted into aglycones by the removal of organic acids, glucuronides, and glycosides [22]. Individual variability in gut microbial activity impacts the bioavailability of polyphenols, which typically reach the colon and interact with microbes [23]. Rather than liver metabolism, this interaction is the primary source of interindividual variation [24]. Despite numerous studies, no clear relationship has been established between polyphenol bioavailability and bioactivity. The gut microbiome’s role in polyphenol conversion leads to delayed metabolite appearance in circulation and significant variation in absorption rates and levels [25]. These factors must be considered in future research designs to better understand and utilize the microbiota’s polyphenol bioconversion capacity.

The influence of non-polyphenolic dietary components on microbial bioconversion must be considered to standardize and control the background diet in pharmacokinetic studies [26]. In this study, the polyphenol content of the background diet was carefully controlled and healthy volunteers were consistently provided with curd, butter, and eggs during all meals to maintain dietary consistency. However, this non-polyphenolic diet may have affected regular pharmacokinetics [27]. The low-phenolic diet was provided based on previous research. As no established guidelines exist for controlling diet, water intake, physical activity, and blood withdrawal during herbal drug pharmacokinetic studies, conventional methods were used to standardize these variables at different time points.

Various foodstuffs can affect drug pharmacokinetics and pharmacodynamics by influencing oral bioavailability, transport, metabolism, and systemic distribution, ultimately, impacting clinical efficacy [28]. Despite normal protein levels, dietary proteins consumed during the study may have interacted with the drug’s kinetic profile. Given the constraints of human study design, fully exploring this interaction is challenging. However, research suggests multilevel approaches can effectively distinguish subtle polyphenol-induced effects amid large phenotypic variations in human subjects [29].

Limitations

The primary limitation of this study was the unavailability of adequate resources to evaluate gallic acid metabolites in blood and urine. Due to constraints in the facility, we could not standardize methods for accurate metabolite estimation. Additionally, limited resources affected the sample size and the ability to control external variables, which may have influenced the results. These resource-related challenges highlight the need for further research with enhanced facilities to obtain more comprehensive data.

## Conclusions

This study evaluated the oral pharmacokinetics of the Siddha polyherbal formulation MMC using gallic acid as a biomarker. The observed variations in plasma gallic acid peaks across participants highlight significant individual pharmacokinetic differences, likely influenced by factors such as pharmacogenomics, gut microbiota, and dietary interactions. Despite normal serum protein and albumin levels, dietary proteins may have impacted the drug’s kinetic profile. A key strength of this research is the novel application of personalized pharmacokinetics, which considers individual Siddha body types, underscoring the importance of tailored approaches in the pharmacokinetic evaluation of herbal formulations.
